# Breath Alcohol Concentration and Perception of Drunkenness: A Comparison between Adolescents and Young Adults Drinking Alcohol in Public Settings

**DOI:** 10.3390/ijerph17082890

**Published:** 2020-04-22

**Authors:** Elena Gervilla, Rafael Jiménez, Joella Anupol, Mariàngels Duch, Albert Sesé

**Affiliations:** 1 Balearic Islands Health Research Institute, University of the Balearic Islands, 07122 Palma, Spain; elena.gervilla@uib.es (E.G.); albert.sese@uib.es (A.S.); 2 European Institute of Studies on Prevention, 07003 Palma, Spain; janupol@irefrea.org (J.A.); mduch@irefrea.org (M.D.)

**Keywords:** alcohol, adolescent, breath alcohol concentration, drunkenness, self-reported, public setting

## Abstract

Alcohol use is a persisting social and health problem in Spain that often takes place within the recreational context. This study aims to analyze objective and self-reported measures of alcohol use and to assess the potential role of social factors on alcohol intake in open-air public settings. A total of 1475 participants (47.4% women), including 27.8% adolescents organized into 355 natural groups of friends, were interviewed while they were socializing at night in the streets of Palma (Spain). Breath alcohol concentration (BrAC), self-reported measures of alcohol use, and social variables were assessed. Men showed statistically higher scores in BrAC than women. However, adolescents’ Alcohol Use Disorders Identification Test (AUDIT) scores were not statistically different by gender. Correlation between objective and self-reported measures was low. Interestingly, BrAC of drinkers was lower when some friends in the group were sober. Moreover, especially in young adults, variables related to the social environment were statistically significant factors to predict BrAC. In conclusion, we found a high prevalence of alcohol intake in young people in open-air public settings, low relationship of objective and self-reported measures, and social factors linked to alcohol use, although differences by age and gender must be considered.

## 1. Introduction

Adolescents initiate alcohol use in a stage when development has not yet finished. In Spain, 13.2% people aged 15–24 drank alcohol in public settings [1]. Moreover, 58.5% of adolescents consumed alcohol in the last month, and almost a quarter (23.7% boys and 25.0% girls) engaged in acute alcohol intoxication in the last month [2]. The European School Survey Project on Alcohol and Other Drugs [3] indicates that the European average proportion of alcohol intoxication among 16 year-olds is 13%, while in Spain this average proportion reached 21%. This behavior places them at risk for negative consequences, especially during this developmental stage [4,5,6].

Measurement of alcohol use often relies on self-reported measures (i.e. number of alcoholic drinks used, perception of drunkenness). However, self-reported measures can be inaccurate when they are collected in drinking environments, particularly among younger drinkers since situational factors can affect them [7]. Further, the exposure to other people’s drinking may be shaping the perception of context-related drinking norms [8,9,10,11,12]. Moreover, perception of drunkenness is also related to the intoxication level of other members in the group, mainly those in the same gender group [13]. Studies that assess alcohol use with an objective measure have also found that drinkers in naturalistic settings inaccurately estimate their intoxication [7,14].

On the other hand, it seems that gender and age also have an influence in the accuracy of alcohol use estimation. In this sense, it has been found that females and participants under 21 years of age tend to underestimate their breath alcohol concentration (BrAC) [15], although some authors consider that the influence of gender on BrAC estimation needs further research [16]. 

The aim of this study is (a) to describe alcohol use and perception of drunkenness in open-air public settings, (b) to analyze the relationship between objective and subjective measures of alcohol use, and (c) to assess the influence of individual and contextual factors on alcohol use.

## 2. Materials and Methods

A total of 1475 participants (organized into 355 groups of friends) who were participating in *botellon* (gatherings of binge-drinkers in open-air) in public settings in the city of Palma (Balearic Islands, Spain) accepted to be anonymously interviewed (15.3% declined to participate). Convenience sampling was used to select participants. Interviewers collected information about the public settings where young people were going to meet and went there to interview them.

Participants had a median age of 21 years (interquartile range = 5) and 47.4% were women. The World Health Organization defines ‘Youth’ as the 15–24-year age group and ‘Adolescents’ as individuals in the 10-19-year age group. Following WHO classification, we found 27.8% adolescents in our sample. Moreover, 8.5% were under the minimum age limit for alcohol drinking in Spain which is 18 years. Table 1 shows the proportion and number of males and females in each age group.

Upon recruitment, participants completed a survey, which included a brief interview about sociodemographic factors (gender, age), perception of drunkenness (Likert scale, 1 being totally sober and 10, totally drunk), number of alcoholic beverages drunk, and consumption of food in the previous 2 h. In order to have an objective measurement of alcohol concentration, we assessed breath alcohol concentration (BrAC) (mg/L) with a breathalyzer (Zaphir 3500BT, Consumer Design Products, Madrid, Spain). Regarding social factors, we calculated the proportion of peers in the natural group of friends who had BrAC = 0 and we created a dichotomous variable indicating whether all the members in the group of friends had a BrAC > 0 or some member had a BrAC = 0. Participants were also asked to answer the alcohol use disorders identification test (AUDIT) [17]. Finally, we calculated the amount of time that had passed between first drink and the assessment of BrAC. Information was collected through the Limesurvey mobile app [18].

Recruitment occurred on Thursday, Friday, and Saturday nights. All participants provided verbal informed consent. Ethical approval was granted by the ethics committee of University of Balearic Islands (approval number 75CER18). Between 11 p.m. and 3.30 a.m., teams of 2–5 researchers randomly selected the groups to be interviewed. Before assessing the BrAC, participants were asked to rinse their mouth with water to eliminate any residual alcohol traces. There were no financial incentives for participation.

Before approaching participants, researchers visually assessed their level of drunkenness based on three visual indicators of intoxication: how steady they were on their feet, whether they were swaying, and how loud they were talking [19]. Special attention was given to ethical concerns on interviewing intoxicated individuals. Interviewers were instructed not to approach individuals who appeared to be extremely intoxicated or aggressive. Furthermore, members of the research team were trained to call the emergency services when a person showed signs of alcohol poisoning and to help them, especially underage participants, if they had drunk. Finally, participants who had a BrAC higher than the Spanish legal limit (0.25 mg/L) were told not to drive.

## 3. Results

### 3.1. Breath Alcohol Concentration and AUDIT Scores

Regarding alcohol use, it is worth noting that most of the sample (80.2%, n = 1183) had a BrAC score higher than 0 when they were interviewed. Sample demographics and BrAC scores of participants with BrAC higher than 0 are listed in Table 2. Statistics show that the distribution of the variable does not fit the normal distribution. Participants had a BrAC median score of 0.23.

Comparison of people with BrAC = 0 and people with BrAC > 0 in terms of individual characteristics can be observed in Table 3. It is remarkable that men and women were not equally represented in the two groups: we found more women with BrAC = 0 and more men with BrAC > 0. Moreover, participants with BrAC > 0 were older and most of them were employed. It is worth noting also that 24.5% of participants with BrAC > 0 were adolescents and 7% of participants with BrAC > 0 did not have the minimum age limit for alcohol drinking in Spain.

We found statistical differences by gender and age in BrAC: men had higher alcohol concentration scores (mg/L) than women, regardless of their age, and adolescents presented lower BrAC than people above 19 years old (see Table 4). However, there was no statistically significant difference in the mean BrAC of participants under and above the minimum age limit for alcohol drinking in Spain.

**Figure 1:**
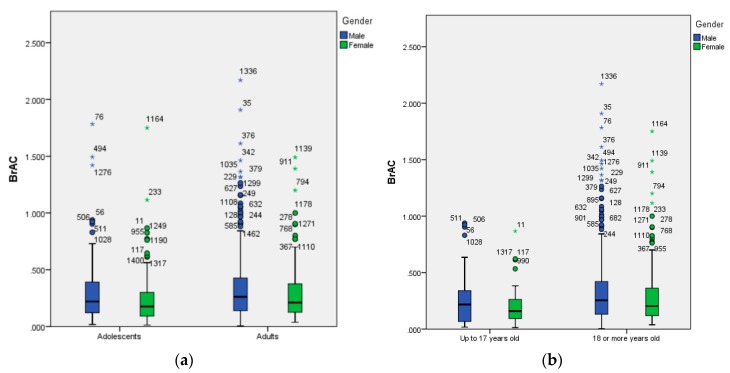
(**a**) Boxplot of BrAC by chronological age (adolescents and young adults) and gender (male and female); (**b**) boxplot of BrAC by age limit for alcohol drinking in Spain (up to 17 years old and 18 or more years old) and gender (male and female).

Figure 1 shows the graphic representation through boxplot of BrAC scores by age and gender. Using robust statistics, we can also see that men showed higher BrAC scores than women, regardless of the age group. Moreover, there is a remarkably high proportion of outliers, especially in male adults.

Regarding AUDIT scores, men also showed higher scores than women in the adults’ sample. However, we did not find statistical differences in AUDIT scores by gender among the adolescents’ group, which indicates that adolescent boys and girls showed the same scores in the screening of risky alcohol use. Finally, adults had higher scores than adolescents in the AUDIT score (see Table 5), while this difference was not statistically significant if we compare AUDIT scores of participants under and above the age limit for alcohol drinking in Spain.

**Figure 2:**
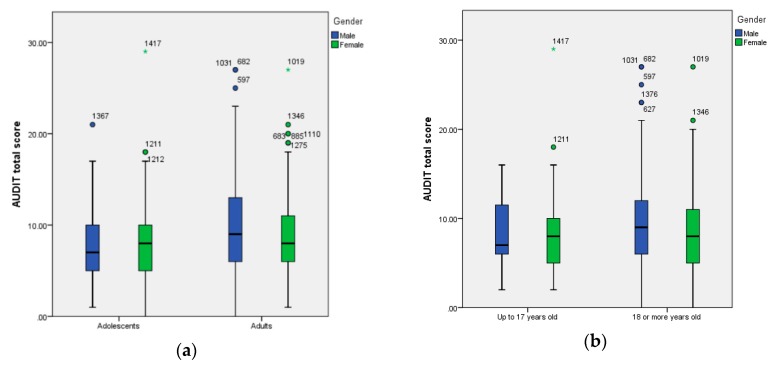
(**a**) Boxplot of AUDIT total scores by age (adolescents and young adults) and gender (male, female); (**b**) boxplot of AUDIT total scores by age limit for alcohol drinking in Spain (up to 17 years old and 18 or more years old) and gender (male and female).

Figure 2 shows the graphic representation through boxplot of AUDIT scores by age and gender. Using robust statistics, we can also see a different pattern by age: while males show higher scores than females among the sample of adults, females present higher scores than males in the underage sample (b). 

### 3.2. Relationship Between Objective and Self-Reported Measures

To analyze if there was a relationship between objective and self-reported measures of alcohol use, we ran correlations between these two factors for those participants with BrAC > 0 and we controlled by the amount of time they have spent in the public setting. Results showed that correlations were low but statistically significant (see Table 6). Accordingly, coefficients of determination (R^2^) are weak in all the subsamples.

### 3.3. Influence of Contextual Factors on Alcohol Use

In order to assess the influence of contextual factors on alcohol use, we also collected objective and self-reported information on the group of friends. The comparison of BrAC means in groups formed by all members with a BrAC > 0 and groups with sober components indicated that BrAC scores decrease when some friends in the group are sober. Hypothesis testing showed that the mean difference remained statistically significant (see Table 7). In order not to underestimate the average value of BrAC in the group of friends, we eliminated the values for sober people. So, average BrAC in the group represents the value of BrAC only for those who had BrAC > 0.

**Figure 3:**
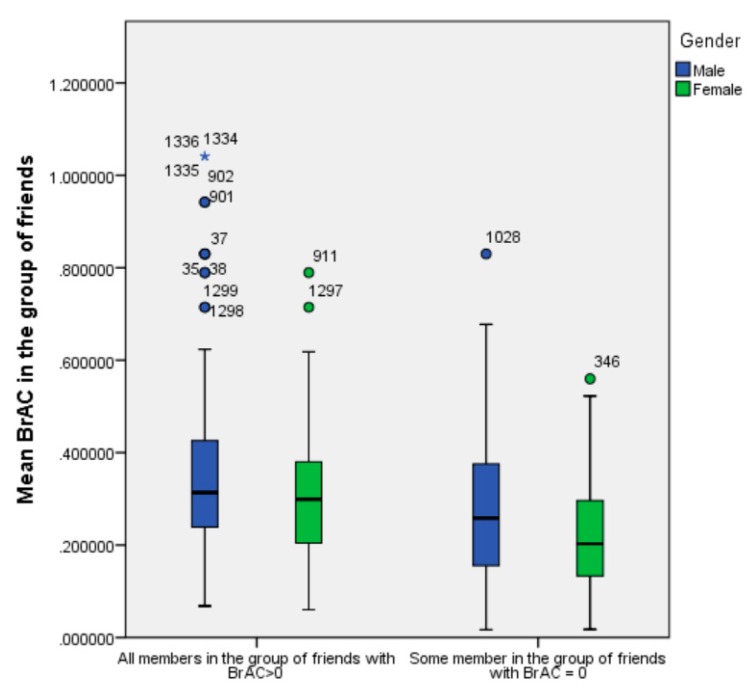
Boxplot of BrAC by proportion of friends with a BrAC > 0 (all members with BrAC > 0 and some members with BrAC = 0) and gender (male, female).

Figure 3 shows the boxplot graphic representation of the average BrAC scores in the group of friends by gender and participation or absence in the group of sober members. Using robust statistics, we can observe that, when all members in the group had BrAC > 0, the average of alcohol concentration in the group was higher than the average BrAC score for groups where member/some members was/were sober. The high proportion of outliers was remarkable, especially in men with all friends with BrAC > 0.

Finally, to assess the influence of individual and contextual factors on alcohol use, multiple linear regressions were calculated to predict BrAC. Factors assessed in this study include the proportion of sober people in the group, gender, age, AUDIT score, proportion of peers with a risk score in AUDIT, number of alcoholic drinks consumed and intake/ingestion of food in the previous two hours. We ran the models separately for adolescents and young adults, controlling by the amount of time they had spent in the public setting. Results show that among young adults, a significant regression equation was found (F (8, 604) = 29.649; *p* < 0.001), with an adjusted R^2^ of 0.272. Young adults’ predicted BrAC is shown in Equation (1), highlighting social (proportion of sober friends and proportion of peers with risk AUDIT) and individual (number of alcoholic drinks consumed) variables as relevant in the estimation of BrAC scores:

BrAC = 0.311 − 0.291 (proportion of sober friends) + 0.168 (minutes in public setting) + 0.216 (AUDIT total score) − 0.103 (proportion of peers with risk AUDIT) + 0.143 (number of alcoholic drinks consumed).(1)

The model was also statistically significant for adolescents (F (8,54) = 3.278; *p* = 0.004) with an adjusted R^2^ of 0.227. Adolescents’ predicted BrAC is shown in Equation (2) and indicates that only the proportion of sober friends is a relevant variable to predict BrAC:

BrAC = 0.192 − 0.263 (proportion of sober friends) + 0.209 (minutes in public setting).(2)

The model was also statistically significant for participants under the minimum legal age to drink in Spain (18 years of age) (F (8,220) = 6.549; *p* < 0.001) with an adjusted R^2^ of 0.163. However, only the intake/ingestion of food in the previous two hours was a statistically significant factor to predict BrAC in this subgroup.

## 4. Discussion

The aim of this study was to describe alcohol use and perception of drunkenness in public settings, to analyze the relationship between objective and subjective measures of alcohol use, and to assess the influence of social factors on alcohol use. We found that more than 80% of people participating in *botellon* in open-air public settings at night were drinking alcohol, more than a quarter of them were adolescents and 7% of those who were drinking did not have the legal age to use alcohol in Spain. Males had higher alcohol concentration scores (mg/L) than females, and adults showed higher values than adolescents. Regarding AUDIT scores, we found that males had higher scores than women among adults, while underage girls showed higher scores than boys. In this sense, it is worth noting the high mean values for AUDIT scores found in participants, being all of them above traditional risk thresholds [20] and the psychosocial and health consequences that this risk alcohol use may be linked with, especially in females.

Regarding the relationship between objective and subjective measures of alcohol use, it is noteworthy that they were low. Our results are in line with previous studies assessing alcohol use with an objective measure which have found that participants tend to underestimate their breath alcohol concentration [15]. Some authors have shown that the perception of the effects of alcohol in terms of drunkenness seems to vary across countries [21]. Future studies may assess if these results vary depending on local culture.

Finally, in line with other works [11,12,13,14,22,23,24] our results highlight the relevance of social variables on alcohol use, although it seems that age plays an important role on this relationship. In this sense, having sober friends is associated with a reduced breath alcohol concentration, especially in adolescence. Moreover, in young adults, social (proportion of sober friends and proportion of peers with risk AUDIT) and individual (number of alcoholic drinks consumed) variables were statistically significant factors to predict BrAC. However, only the proportion of sober friends in the group was a statistically significant factor to predict BrAC in the subgroup of adolescents.

Future studies should analyze if people pre-drinking in open-air public settings have higher or lower consumption when moving to a venue later on. Some research has shown that, even if the reason posed for drinking in public settings is to save money when moving to a venue pre-drinkers tend to show higher consumption rates [12]. Future research may also assess if some individual factors might influence others to drink in a social drinking context. In this sense, some research has found that members of a higher status engaged in the most alcohol consumption only in heavier drinking groups [25].

Our study has some limitations. The list of items assessed in the survey was limited by the characteristics of the natural setting and, therefore, we have not assessed some factors that could potentially contribute to alcohol use, namely personality factors. Moreover, participants could have completed the assessment on more than one occasion, which limits the generalizability of the findings. Due to the cross-sectional perspective of this study, we cannot assess if peer influence or selection processes have formed the group of friends. However, reciprocal influences have been found in adolescence between drinking and frequently being chosen as a friend [26]. Finally, factors that can influence alcohol absorption (i.e., the pattern of drinking throughout the night, tolerance) were not included in the questionnaire.

## 5. Conclusions

The current study advances knowledge of the relationship between subjective drunkenness and objective assessment of intoxication and the role of social factors on alcohol use in a natural setting, with a big sample of participants including adolescents. These results support the emphasis on peer influence and contextual factors in preventive programs. 

## Figures and Tables

**Table 1:** Proportion and number of males and females in each group of age.

		Male	Female
Developmental age	Adolescents	38.8% (n = 159)	61.2% (n = 251)
	Adults	57.9% (n = 616)	42.1% (n = 448)
Legal age to drink alcohol	Up to 17 years old	37.3% (n = 47)	62.7% (n = 79)
	18 years old or more	54.0% (n = 728)	46.0% (n = 620)

**Table 2:** Sample demographics and BrAC scores of participants who had a BrAC score higher than 0.

Statistics (n = 1183)	BrAC (mg/L)
Mean (SD)	0.2928 (0.25)
Median (IQR)	0.2300 (0.26)
Skewness (SE)	2.403 (0.071)
Kurtosis (SE)	9.330 (0.142)

**Table 3:** Comparison of people with BrAC = 0 and people with BrAC > 0 in terms of Individual Characteristics.

	BrAC = 0 (n = 292)	BrAC > 0 (n = 1183)	Hypothesis Testing
Gender (Female)	55.5%	45.4%	*χ*^2^ = 9.556; df = 1; *p* = 0.002
Age (M, SD)	20.73 (4.10)	22.25 (4.19)	*t* = −5.576; df = 1472; *p* < 0.001
Employed	41.1%	56.5%	*χ*^2^ = 22.310; df = 1; *p* < 0.001
Adolescents	41.1%	24.5%	*χ*^2^ = 31.986; df = 1; *p* < 0.001
Under the minimum age limit for alcohol drinking in Spain (18 years of age)	14.7%	7.0%	*χ*^2^ = 17.778; df = 1; *p* < 0.001

**Table 4:** Comparison of BrAC by gender and age (for participants with BrAC > 0).

BrAC Scores (mg/L)		M (SD)	Hypothesis Testing
Adults	Male (n = 532)	0.33 (0.27)	*t* = 3.405; df = 879.976; *p* = 0.001
	Female (n = 360)	0.27 (0.20)	
Adolescents	Male (n = 113)	0.30 (0.30)	*t* = 2.240; df = 184.011; *p* = 0.026
	Female (n = 177)	0.23 (0.21)	
Age	Up to 19 years old (n = 290)	0.26 (0.25)	*t* = −2.566; df = 1180; *p* = 0.010
	Above 19 years old (n = 892)	0.30 (0.25)	
Under the minimum age limit for alcohol drinking in Spain (18 years of age)	Up to 17 years old (n = 83)	0.24 (0.22)	*t* = −1.913; df = 1180; *p* = 0.056
	18 years old of age or more (n = 1099)	0.30 (0.25)	

**Table 5:** Comparison of AUDIT scores by gender and age (for participants with BrAC > 0).

AUDIT Scores		M (SD)	Hypothesis Testing
Adults	Male (n = 309)	9.87 (4.67)	*t* = 3.157; df = 524; *p* = 0.002
	Female (n = 217)	8.59 (4.43)	
Adolescents	Male (n = 61)	8.03 (4.14)	*t* = −0.075; df = 164; *p* = 0.941
	Female (n = 105)	7.98 (4.41)	
Age	Up to 19 years old (n = 166)	8.00 (4.30)	*t* = −3.329; df = 690; *p* = 0.001
	Above 19 years old (n = 526)	9.34 (4.61)	
Under the minimum age limit for alcohol drinking in Spain (18 years of age)	Up to 17 years old (n = 42)	8.62 (5.18)	*t* = −0.590; df = 690; *p* = 0.555
	18 years old or more (n = 650)	9.05 (4.54)	

**Table 6:** Pearson correlation between BrAC scores and perception of drunkenness (in participants with BrAC > 0 and controlling by minutes in drinking environment) and proportion of participants who indicated the different answers in perception of drunkenness.

BrAC - Perception of Drunkenness			Perception of Drunkenness (%)									
	R	R^2^	1	2	3	4	5	6	7	8	9	10
Total sample (df = 1115)	0.259 **	0.0670	21.7	17.9	17.2	13.3	9.6	11.5	6.6	1.8	0.2	0.2
Women (df = 510)	0.265 **	0.0671	26.8	20.0	17.0	12.0	8.4	7.7	5.4	2.1	0.2	0.4
Men (df = 602)	0.241 **	0.0581	17.4	16.2	17.4	14.5	10.6	14.6	7.6	1.6	0.2	0
Adolescents (df = 271)	0.240 **	0.0576	26.0	14.9	14.5	14.2	7.6	11.8	6.6	3.5	0.7	0.3
Under the minimum age limit for alcohol drinking in Spain (18 years of age) (df = 74)	0.276 **	0.0762	30.1	8.4	12.0	15.7	7.2	12.0	4.8	7.2	1.2	1.2

** *p* < 0.001.

**Table 7:** Comparison of BrAC by number of peers drinking (for participants with BrAC > 0).

BrAC Scores (mg/L)	Mean (SD)	Hypothesis Testing
All members with BrAC > 0 (n = 687)	0.3219 (0.1459)	*t* = 8.331; df = 1165; *p* < 0.001
Some members with BrAC = 0 (n = 480)	0.2509 (0.1391)	
